# Vaccinations and Autoimmune Diseases

**DOI:** 10.3390/vaccines9080815

**Published:** 2021-07-22

**Authors:** Bianca Olivieri, Corrado Betterle, Giovanna Zanoni

**Affiliations:** 1 Department of Medicine, School of Specialization in Allergy and Clinical Immunology, University of Verona, 37134 Verona, Italy; bianca.olivieri@studenti.univr.it; 2 Department of Medicine (DIMED), Clinical Immunology and Allergy, University of Padua, 35128 Padua, Italy; corrado.betterle@unipd.it; 3 Immunology Unit, University Hospital, 37134 Verona, Italy

**Keywords:** vaccines, autoimmunity, autoimmune diseases, Guillain-Barré syndrome, thrombocytopenia, SARS-CoV2 vaccines, vaccine-induced immune thrombotic thrombocytopenia

## Abstract

Vaccines represent one of the most effective measures of public health medicine, saving countless lives and preventing lifelong disabilities. Vaccines are extremely safe, however, no vaccine is completely free from risks and adverse events can occur following vaccination. An adverse event following immunization (AEFI) may be a true adverse reaction caused by the vaccine or an event that temporally occurred after immunization but is not caused by it. Among the adverse reactions to vaccines, one of the most feared is the triggering of autoimmune diseases, which are a heterogeneous group of disorders characterized by dysregulation of the immune system. Currently, no mechanisms have been demonstrated that could explain the correlation between vaccination and the development of autoimmune diseases. Furthermore, epidemiological studies do not support the hypothesis that vaccines cause systemic autoimmune diseases. The only confirmed associations, although very rare, are those between the flu vaccine and Guillain-Barré syndrome, especially with old vaccine preparations, and measles-mumps-rubella (MMR) vaccine and thrombocytopenia. Due to the SARS-CoV2 pandemic, new types of vaccines have been developed and are now available. Close vaccine safety-surveillance is currently underway for these new vaccines.

## 1. Introduction

Immunization is one of the most effective measures of public health medicine, saving countless lives and preventing lifelong disabilities. Vaccines are extremely effective, highly safe, and are mostly affordable; therefore, they have allowed not only a reduction in the incidence of infections, but also a reduction in the mortality and morbidity related to them [1].

Vaccines are often obtained from weakened or killed forms of the microbe, its toxins, or its surface proteins. Traditionally, there are four types of vaccines: live-attenuated, inactivated (killed), subunit (purified antigen) and toxoids (inactivated toxic compounds) [2]. Due to the SARS-CoV2 pandemic, other types of vaccine have been developed by new technologies and are now available, such as the mRNA vaccines or the virus-vectored vaccines [3]. Vaccines may contain, in addition to the antigen, other components, such as adjuvants, capable of enhancing the immune response to the antigen; stabilizers, which allow the characteristics of the preparation to be maintained; and preservatives, sometimes including traces of residual antibiotics, used to prevent bacterial or fungal contaminations during production process [2].

National immunization programs offer extremely safe and effective vaccines. However, no vaccine is completely free of risk and adverse reactions will occasionally occur following vaccination. An adverse event following immunization (AEFI) is defined by the World Health Organization (WHO) as “any untoward medical occurrence which follows immunization, which does not necessarily have a causal relationship with the usage of the vaccine” [2]. Reported AEFI may be proven adverse reactions, caused by the vaccine or the immunization process, or events that temporally occurred after immunization but are not caused by it. AEFI can be classified into five categories based on the specific cause that resulted in the adverse event: vaccine product-related reaction, vaccine quality defect-related reaction, immunization error-related reaction, immunization anxiety-related reaction and coincidental event. AEFI can range from mild reactions, which are the majority and are very often self-limiting, to serious although rare reactions [4]. Causality assessment is meant to assist in determining the level of certainty of an association between the immunization and an AEFI, which may be classified as consistent, inconsistent, or indeterminate. Causality assessment of individual reports is performed according to WHO classification by a committee of reviewers [5]; when necessary, epidemiological studies are planned to investigate potential safety signals. Vaccine safety is continuously monitored through post-marketing surveillance conducted at multiple levels by the pharmacovigilance sections of various institutions, such as WHO [5] and its Global Advisory Committee on Vaccine Safety (GACVS), the European Medicines Agency (EMA) in Europe [6], the Centers for Disease Control and Prevention (CDC) and the Food and Drug Administration in the United States, and the national vaccine surveillance systems in each country.

Adverse reactions caused by vaccines are much lower in frequency and severity than those caused by spontaneous infection. The majority of adverse reactions are mild, such as injection site pain or fever, and only a small number of cases may be very serious or require medical attention [7,8]. Despite significant benefits of vaccination, misconceptions about vaccine safety still affect public confidence and hesitancy can reduce adherence to immunization programs [9]. Among the adverse reactions to vaccines, one of the most feared is the triggering of autoimmune diseases [10].

Autoimmune diseases are a group of heterogeneous diseases characterized by an aberrant immune response caused by the loss of self-tolerance. These conditions can be systemic or organ-specific, but all of them have an etiology that is not fully understood. Infectious agents, particularly viruses, along with other environmental, gender and other endogenous factors, genetic and epigenetic factors, seem to participate in their pathogenesis [11,12,13,14]. Various mechanisms have been proposed to explain the role of microorganisms in the pathogenesis of autoimmune diseases, such as molecular mimicry, epitope spreading, bystander activation and polyclonal activation [15].

Vaccines have long been suspected to play a role in the development of autoimmune diseases. The main hypothesis that has been proposed to explain this immunological association is epitope mimicry, just like in infections. According to this mechanism, an antigen that is administered with the vaccine may share structural similarities with self-antigens. The immune response to the vaccine antigen could therefore also extend to other host cells expressing the structurally similar self-antigen. A second mechanism that could be involved is bystander activation. It is an antigen non-specific mechanism that leads to the activation of autoreactive T cells [16]. However, the pathogenetic mechanisms that explain the causal link between vaccinations and autoimmune diseases are not yet fully understood and they are also difficult to study [17]. 

Except for the rare associations between flu vaccine and Guillain-Barré syndrome and between measles-mumps-rubella vaccine (MMR) and thrombocytopenia, the role of vaccines in the development of autoimmune diseases has not been established [18,19]. Many isolated cases or case series of arthritis, vasculitis and central or peripheral nervous system symptoms in temporal relationship with vaccination are reported as AEFI in the scientific literature; despite this, currently no mechanisms have been demonstrated that can explain the correlation between vaccination and the development of chronic autoimmune diseases. Furthermore, epidemiological studies do not support the hypothesis that vaccines cause systemic autoimmune diseases [10,20].

In this review, we are going to analyze the available evidence regarding vaccines mostly evaluated in regard to potential risks of inducing autoimmune diseases. Furthermore, we are going to highlight the key points of vaccination management in the patient affected by a systemic autoimmune disease. Finally, we are going to report the latest available data on autoimmune reactions following vaccination with anti-SARS-CoV2. 

## 2. Live Attenuated Vaccines: Measles-Mumps-Rubella and Varicella-Zoster

Vaccine-preventable viral diseases can cause serious complications and rarely death. 

Measles can lead to complications such as diarrhea (6%), otitis (7–9%), pneumonia (1–6%), post-infectious encephalomyelitis (0.5/1000) and subacute sclerosing panencephalitis (1/100,000). About 15% of subjects affected by encephalitis die and 25% have brain injuries [2].

Mumps can cause severe complications: aseptic meningitis (10%), pancreatitis (4%), encephalitis (0.06–0.3%), deafness (0.007%), orchitis in up to 38% males and oophoritis in up to 5% in females. Mortality is estimated at 0.02%. If the infection is contracted during the first trimester of pregnancy, there is an increased risk of miscarriage [2].

Rubella is usually a mild disease, but if this infection occurs in the first eight weeks of pregnancy, up to 85% of newborns may have one or more permanent defects, such as deafness, blindness, brain damage and heart problems [2]. Post-natal rubella has been correlated with thrombocytopenic purpura [21], especially in children, but also with myocarditis [20] or chronic arthritis [22] in cases of persistence of the virus.

Varicella zoster virus (VZV) infection, also called “chicken pox”, is more common in children under 10 years of age; secondary or recurrent infection is caused by reactivation of the virus and is also called “shingles”. Chickenpox infection can cause even serious complications, such as pneumonia, aseptic meningitis or encephalitis, transverse myelitis and Guillain-Barré syndrome, myocarditis, arthritis, orchitis, uveitis, iritis and hepatitis [2].

The availability of live attenuated measles, mumps and rubella vaccines has almost completely eliminated serious neurologic complications of measles and dramatically reduced cases of congenital rubella [23,24,25]. These vaccines are currently administered in trivalent MMR vaccine. Varicella vaccine can be single antigen or combined with measles, mumps, rubella and varicella (MMRV). The most significant adverse event reported following MMR vaccination is thrombocytopenic purpura, which is consistent with the immune-mediated manifestations documented after wild virus infections, although much rarer. After MMR vaccination, there have also been very rare cases of anterior uveitis [26,27], retinopathy [28], vasculitis, and myositis [20,29]. Several AEFI have been reported to the US Vaccine Adverse Events Reporting System (VAERS) after varicella vaccination: cases of encephalitis, aseptic meningitis, facial paralysis, arthritis, thrombocytopenia, vasculitis, erythema multiforme and, more rarely, cases of optic neuritis, transverse myelitis, Guillain-Barré syndrome. However, this passive surveillance system records data of suspected adverse reactions but does not assess causal relationship [20,30,31].

Idiopathic thrombocytopenic purpura (ITP) has been confirmed as a rare adverse reaction after MMR vaccination [32,33]. The median time to onset of thrombocytopenia is 12–25 days after immunization, but the range is 1–83 days [2]. However, the risk of ITP after vaccination is smaller than after natural infection with these viruses. In fact, the attributable risk is estimated to be about 1 case of thrombocytopenia per 40,000 administered MMR doses, while thrombocytopenia after natural rubella infection occurs in 1 case per 3000 [34,35]. Most episodes of ITP following immunization resolve within three months and rarely does thrombocytopenia persist for up to six months [36].

Episodes of transient arthralgia (in 25% of cases) and acute arthritis following MMR vaccination are reported. These joint manifestations are due to the rubella virus component present in the MMR vaccine [2]. Acute arthritis onset usually occurs within six weeks of immunization; after this period it is unlikely that arthritis is related to the vaccine [37]. Female gender, older age, previous seronegativity and particular HLA types may be risk factors. However, there is no evidence of increased risk for chronic arthropathy among women vaccinated against rubella [20,38,39,40]. A recent systematic review has pointed out that the reported cases of arthritis after vaccinations are too heterogeneous and incomplete to confirm a causal association with the administered vaccine [41].

MMR-related aseptic meningitis, which typically resolves spontaneously within approximately one week without permanent sequelae, has been reported. Prior to 1989, epidemiological studies had revealed that vaccination-related mumps meningitis occurred after less than 1 in 100,000 vaccine doses. An increase in cases of aseptic meningitis has been reported since 1989 with varying frequencies in different countries. This increase was due to the presence of UrabeAM9 mump strain. Urabe-containing vaccines were withdrawn from the market after this finding [42]. Currently there is no evidence of an association between MMR immunization and aseptic meningitis, encephalitis and encephalopathy. Furthermore, there is also no causal correlation between MMR vaccine and autistic spectrum disorders [34]. 

At present, it has not been possible to demonstrate a statistically significant association between MMR vaccination and Guillain-Barré syndrome. However, there are data that seem to indicate that, if there is a risk, it is so low that it cannot be demonstrated by epidemiological studies comparing the incidence of Guillain-Barré syndrome between periods with or without the use of the MMR vaccine, even on large population numbers [43,44,45]. Moreover, there is insufficient evidence to determine the association between MMR immunization and inflammatory bowel diseases. There is no evidence to support an association between MMR immunization and type 1 diabetes mellitus (DM-1), and multiple sclerosis according to a recent meta-analysis [34].

Zoster vaccine is a live attenuated vaccine recommended for older adults to reduce the incidence of herpes zoster and its complication of postherpetic neuralgia. A case-control study of reported events to the VAERS showed no significantly increased risk of severe autoimmune events after vaccination, except for arthritis and alopecia (respectively 2.2 and 2.7 time the odds compared to unexposed subjects). The authors concluded that zoster vaccine is relatively safe and unlikely to exacerbate or induce autoimmune diseases [46].

## 3. Recombinant DNA Vaccine: Hepatitis B 

Hepatitis B is an infectious disease caused by the hepatitis B virus (HBV) that affects over 350 million individuals worldwide and that is associated with the development of cirrhosis (5%) and hepato-carcinoma (5%) [2]. Chronic hepatitis B infection is associated with extrahepatic manifestations, mostly immune-mediated and in particular associated with the formation of immune complexes. These include skin rash, arthralgias and arthritis, polyneuritis, glomerulonephritis and polyarteritis nodosa (PAN) [47,48].

The frequency of autoimmune manifestations reported after administration of the HBV vaccine, based on recombinant DNA technology to express HBsAg, is extremely low compared to the tens of millions of vaccinations performed [20]. Cases of arthritis, rheumatoid arthritis (RA) [49,50], thrombocytopenia [51], vasculitis [52], demyelinating encephalitis [53], and other neurological manifestations have been reported [54,55]. There have also been rare reports of Sjögren’s syndrome [56] and cryoglobulinemia [57].

There is currently insufficient evidence to establish a causal link between the HBV vaccine and encephalitis, acute disseminated encephalomyelitis (ADEM), transverse myelitis, optic neuritis, multiple sclerosis, Guillain-Barré syndrome, brachial neuritis, erythema nodosum, reactive arthritis, psoriatic arthritis, rheumatoid arthritis, juvenile idiopathic arthritis, DM-1, fibromyalgia, onset or exacerbation of Systemic Lupus Erythematosus (SLE) and vasculitis [58]. Not only does the scientific literature currently not confirm the causal link between vaccination and vasculitis [59], but it is estimated that the HBV vaccination campaign has reduced the incidence of polyarteritis nodosa (PAN), which is associated with HBV infection in 34% of cases [60].

Independent scientific institutions, such as the WHO and the WHO’s GACVS and the US Institute of Medicine (IOM), have not found a link between HBV vaccination and autoimmune manifestations [61,62].

## 4. Inactivated Vaccines: Influenza 

Influenza is an acute viral respiratory disease that can cause hundreds of thousands of hospitalizations and thousands of deaths during annual winter epidemics [63]. It is estimated that 290,000–650,000 seasonal influenza-associated respiratory deaths (4.0–8.8 per 100,000 individuals) occur annually. Older people have the highest hospitalization and death rates associated with influenza [64,65]. 

There are three serologically distinct type of human influenza viruses: A, B and C. Type A infection is the most frequent and is responsible for most epidemics and pandemics with relative mortality and morbidity. Due to the ability of influenza A and B viruses to undergo a gradual antigenic change in their surface antigens, annual vaccine administration and annual modification of the influenza vaccine strains to be administered is required [2]. There are several types of influenza vaccines, but the most widely used are currently inactivated preparations containing subunit or subvirion (split) surface antigens.

Influenza virus infection can sometimes be implicated in autoimmune complications, such as multiple sclerosis, Guillain-Barré syndrome, epilepsy, DM-1, Schönlein-Henoch syndrome, antiphospholipid syndrome, acute encephalomyelitis, thrombocytopenia and myocarditis [11,66]. After influenza vaccination, cases of vasculitis and rare cases of neurological diseases (e.g., Guillain-Barré syndrome), Schönlein-Henoch syndrome, rheumatoid vasculitis and microscopic polyangiitis have been described [66].

Regarding influenza vaccination and Guillain-Barré syndrome, a sudden increase in cases recorded after the 1976 mass vaccination program in the US was related to the swine influenza A/New Jersey/76 vaccine; therefore, the program was suspended that same year. In fact, 362 cases of Guillain-Barré syndrome occurred following the flu vaccination of 45 million people, with a relative risk of Guillain-Barré syndrome ranging from 4.0 to 7.6 in the period six to eight weeks after vaccination. In the following years, vaccines were prepared with other influenza viruses, resulting in no significant subsequent increase in cases of Guillain-Barrè syndrome [67,68]. A slight increase in the risk of Guillain-Barré syndrome was also recorded for monovalent inactivated influenza A (H1N1) vaccines administered in 2009, with about 1.6 excess cases per million people vaccinated [69]. The association between the influenza vaccine and Guillain-Barré syndrome was closely monitored, noting a slightly increased risk in some seasons but not in others. A meta-analysis of studies published between 1981 and 2014 found that receiving the flu vaccine carried a relative increased risk of Guillain-Barré syndrome of 1.4 [10,70]. On the other hand, in a large retrospective study, there was no increased risk of Guillain-Barré syndrome and its recurrence among pediatric or adult patients within 180 days following vaccinations of any type, including influenza vaccination [71]. In any case, the attributable risk of Guillain-Barré syndrome after influenza vaccination in adults is estimated to be 1–3 in 1,000,000, confirming that it is a very rare event [19]. However, it is important to consider that influenza virus infection carries a greater risk of developing Guillain-Barré syndrome than influenza vaccination. Hence, during an entire flu season, influenza vaccination reduces the risk of developing Guillain-Barré syndrome [72].

Currently there are only case reports of vasculitis or other autoimmune diseases that occur after influenza vaccination on millions of doses administered [73,74,75,76,77]. In particular, 65 patients who developed vasculitis after influenza vaccination were identified from 45 published reports [78]. In a recent study, no significant association was found between influenza vaccination and primary care consultations for disease exacerbations or adverse events in patients with autoimmune diseases [79].

## 5. Vaccines against Invasive Infections by Encapsulated Bacteria: Meningococcal, Pneumococcal, Haemophilus Influenzae Type b Vaccines

Meningitis is a public health problem in most countries, with a morbidity of 1–5 per 100,000 in developed countries and 10–25 per 100,000 in developing countries. The mortality linked to meningococcal meningitis is 5–10%, but reaches 15–20% for fulminant septicemia [2].

There are two types of meningococcal vaccines: meningococcal conjugate (MenC and tetravalent MenACWY135) and serogroup B meningococcal (or MenB) vaccines. During a mass immunization campaign against meningococcus C with conjugated and unconjugated vaccines conducted in France in 2002, the most frequent adverse events were local, neurological and gastrointestinal reactions, mostly transient and not serious. Only 13 serious adverse events were reported, including serum sickness, arthritis, purpura, facial paralysis, multiple sclerosis and meningism. No significant differences were found in the rates of adverse event reports between both vaccines [80]. Immunization with the meningococcal conjugated tetravalent vaccine has not been associated with particular safety concerns or with autoimmune manifestations [81]. Two large studies, which included a total of over 2 million vaccinated adolescents, confirmed that there was no link between meningococcal conjugated vaccination and Guillain-Barré syndrome [82].

Pneumococcal infection, caused by *Streptococcus pneumoniae*, is one of the leading vaccine-preventable causes of death in children in the world, with an estimated mortality of 1.4 per million per year under 5 years of age. Complications of invasive infection include hearing impairment, septicemia, septic arthritis, osteomyelitis, pneumonia and meningitis. Two types of pneumococcal vaccines are available: conjugate vaccines and polysaccharide vaccines. The introduction of the pneumococcal conjugate vaccine has significantly reduced the incidence of pneumococcal infections, including invasive pneumococcal diseases [2].

The conjugate pneumococcal vaccine showed a high level of safety. Rare immune-mediated adverse events (e.g., vasculitis, thrombocytopenia, arthritis or arthralgias etc.) temporally correlated with the administration of the vaccine have been reported to passive pharmacovigilance systems but the causal association remains only hypothetical [83]. Notably, no association was found between 7-valent pneumococcal conjugate vaccine (PCV7) administration in infants and Kawasaki disease [84].

Haemophilus influenzae type b (Hib) is a common cause of bacterial meningitis, pneumonia, and septicemia in children, but can also cause cellulitis (often facial), septic arthritis, and osteomyelitis. It is estimated that it can cause disability with neurological impairment in 15–30% of cases and has a mortality of 5%, with the greatest risk in the second semester of life [2]. 

To date, no significant associations have been reported between Hib and the onset of autoimmune manifestations. A hypothetical increased risk of developing DM-1 in childhood following vaccination [85] was subsequently disproved by further studies [86,87].

## 6. Human Papilloma Virus Vaccines

Human Papilloma Virus (HPV) infection represents the most common sexually transmitted viral infection of the genital tract and is a leading cause of cervical cancer. The morbidity of cervical cancer is about 0.5 million cases per year, while mortality is about 0.25 million cases per year [2].

HPV vaccines are an important means of reducing the incidence of cervical cancer. To date, three types of vaccine are on the market: one nine-valent, one quadrivalent and one bivalent. These vaccines contain non-infectious virus-like particles obtained by recombinant DNA technology [88]. Regarding the quadrivalent vaccine, a post-licensure study was conducted on adverse event reports collected by the VAERS system over a period of 18 months. In total, 51 cases of autoimmune disorders were reported (of which 26 unspecified, 18 of SLE, 13 of RA, 4 of mixed connective tissue disease, 1 of scleroderma, 1 of dermatomyositis, 1 of Sjögren’s syndrome). The authors concluded that most of the adverse events reported were no more frequent compared to those reported after other vaccines [89]. A study has defined quadrivalent vaccine’s impact and safety in the post-licensure surveillance program for use in 20 countries, which has demonstrated general safety, also regarding autoimmunity issues [90]. Another study established that after an observation period of 3 years after vaccination with multiple doses of the HPV vaccine, there was no significant increase in autoimmune diseases in a large proportion of the population [91]. 

Despite the theoretical hypothesis that HPV vaccination may contribute to the onset of autoimmune diseases, recent large-scale studies provide reassuring results. A large population-based cohort study conducted in Denmark and Sweden analyzed more than 696,000 doses of quadrivalent HPV vaccine among females and found no consistent evidence supporting causal associations with several autoimmune and neurologic conditions [92]. These data were also confirmed by a French large case-control study [93]. An analysis of national data from Sweden and Denmark found no increased risk of multiple sclerosis or other demyelinating diseases following HPV vaccination [94]. A meta-analysis that included 20 studies (12 cohort studies, 6 case-control studies, and 2 randomized controlled trials) showed that HPV vaccination was not associated with an increased risk of autoimmune diseases [95]. Another meta-analysis which comprised twenty-two studies revealed that DM-1, immune thrombocytopenia purpura and chronic thyroiditis were the most frequently reported after HPV vaccination. However, this study demonstrated the absence of a clear association between HPV vaccines and autoimmune diseases and other rare diseases [96].

A large cohort study of over 2 million young girls in France reported that the incidence of autoimmune diseases was not increased after exposure to HPV vaccination, except for Guillain-Barré syndrome, with an incidence rate of 1.4 among exposed (20 cases) versus 0.4 per 100,000 per year among unexposed (23 cases) [97]. However, these data have not been confirmed by other studies [98,99]. A recent Australian study examined AEFI from 11 years of post-marketing surveillance. Autoimmune diseases, postural orthostatic tachycardia syndrome, primary ovarian insufficiency, Guillain-Barré syndrome, complex regional pain syndrome and venous thromboembolism, were reported at low rates and analysis did not reveal unexpected patterns that would suggest causal association [99].

## 7. SARS-CoV2 Vaccines

Severe acute respiratory syndrome coronavirus 2 (SARS-CoV2) is an RNA-virus that causes Coronavirus disease 2019 (COVID-19), responsible for the 2020 worldwide pandemic [100]. COVID-19 begins with the viral phase, in which approximately 80% of patients are asymptomatic or with mild symptoms. In the remaining 20% of cases, the disease can be serious and critical, often characterized by a phase of hyper-responsiveness of the immune system. The most serious cases can evolve into a state of hypercoagulability and subsequently organ failure. For this reason, several therapies (i.e., Tocilizumab, Baricitinib, Anakinra etc.), which are usually used in autoimmune inflammatory diseases, have also been tried for the treatment of severe COVID-19, in addition to anti-viral agents and corticosteroids [101]. The immune system appears to play a dual role in SARS-CoV2 infection, both for its activity to control the infection, and its dysregulated response involved in the acute progression of the disease. Therefore it has been hypothesized that this immune dysregulation could induce the loss of tolerance and trigger chronic inflammation [102]. In fact, many cases of autoantibody production, such as anti-nuclear antibodies, have been reported during COVID-19 infection [103]. Various case reports of autoimmune diseases secondary to SARS-CoV2 infection are described in the literature [104], i.e., immune thrombocytopenic purpura [105,106], autoimmune hemolytic anemia [107,108], Guillain-Barré syndrome [109,110,111], Miller Fisher syndrome [112], antiphospholipid syndrome [113,114] and Kawasaki-like disease [115,116]. 

Numerous different technologies are currently being studied and developed in order to create a safe and effective vaccine against COVID-19. The types of vaccines currently on the market are mRNA vaccines and virus-vectored vaccines [117]. Studies currently confirm the overall safety and efficacy of available vaccines [118,119,120,121]. However, rare cases of vaccine-induced immune thrombotic thrombocytopenia (VITT) have been recently reported 5 to 16 days after administration of adenovirus viral-vector vaccines. Most patients were women with a mean age of 36 years (range 22 to 49 years old). VITT consists of severe thrombocytopenia associated with thrombotic phenomena, often in atypical sites, such as cerebral venous thrombosis, splanchnic veins thrombosis, pulmonary embolism, and other types of thrombi. Some patients had more than one thrombotic event at the same time and others had disseminated intravascular coagulation (DIC). In a majority of the patients the outcome was fatal [122,123,124]. In patients with VITT, high levels of antibodies to platelet factor 4 (PF4)–polyanion complexes were identified by enzyme-linked immunosorbent assay (ELISA). In contrast to heparin-induced thrombocytopenia (HIT), binding of antibody to PF4 occurred in the absence of heparin [125]. It is still unclear whether these antibodies are autoantibodies against PF4 induced by the strong inflammatory stimulus of vaccination or vaccine-induced antibodies that cross-react with PF4 and platelets. It is well known that adenovirus is able to bind to platelets and induce their activation, however, it would seem unlikely that the amount of adenovirus in a 500 microliter vaccine injection a week or two earlier would contribute to subsequent platelet activation. Another hypothesis is that the trigger for these PF4-reactive antibodies is free DNA in the vaccine, since both DNA and RNA are capable of forming multi-molecular complexes with PF4 [122].

The risk of venous thromboembolism associated with the vaccines appears to be not higher than the background risk in the general population. These data are important for evaluating the favorable risk-benefit ratio for SARS-CoV-2 vaccines. It is essential that close safety monitoring of these new vaccines continues [126]. 

As for other autoimmune manifestations, case reports of Guillain-Barré syndrome after administration of anti-SARS-CoV2 vaccine have been described [127,128]. However, further studies are needed to evaluate the immune-mediated effects of these new vaccines.

## 8. Adjuvants and Autoimmune Diseases

Various adjuvants are added to vaccines to increase the immune response to specific antigens. Adjuvants can be based on aluminum salts, oils (complete or incomplete Freund’s adjuvant, etc.), virosomes, oil-in-water emulsions and immunomodulatory complexes (such as saponins, ISCOMs); there are also new adjuvants, such as squalene, montanide, lipovant, and xenobiotic adjuvants [129]. Regarding the role of adjuvants in the development of known autoimmune diseases, one study compared pandemic influenza A/H1N1 vaccines that contained adjuvants and those without adjuvants, showing that there was no significant difference in the frequency of reporting of autoimmune manifestations [130].

A hypothetical autoimmune disorder called Autoimmune/autoinflammatory Syndrome Induced by Adjuvants (ASIA) has been proposed [131]. ASIA includes a wide range of different symptoms and conditions which occur after administration of adjuvants (such as silicone, tetramethylpentadecane, pristane, aluminum etc.). Very broad criteria have been codified for the diagnosis of this syndrome [132]. Vaccines containing adjuvants, in particular HBV, influenza and HPV vaccines, have been counted among the supposed various causes of ASIA by the authors who hypothesized the existence of this syndrome [133]. However, a causal relationship between clinical manifestations of the disease and use of vaccine adjuvants is difficult to prove and various studies do not confirm the role of adjuvants in the onset of autoimmune diseases [62,134]. Moreover, the consistency of diagnostic criteria of ASIA syndrome has been questioned in a study conducted on patients undergoing allergen-specific immunotherapy, who received 100 to 500 times more injected aluminum over 3 to 5 years, compared with HBV and HPV vaccine recipients. Patients receiving aluminum-containing allergen immunotherapy preparations had a lower incidence of autoimmune disease [135].

## 9. Vaccinations in Patients with Rheumatic Autoimmune Diseases

Patients with autoimmune inflammatory rheumatic diseases are at increased risk of vaccine-preventable infections, such as influenza, pneumococcal, herpes zoster and HPV infections. For this reason the prevention of these infections is essential in these type of patients [136]. The European League Against Rheumatism (EULAR) recommendations regarding vaccinations in patients with rheumatological diseases have recently been published [137]. Generally, it is preferable to vaccinate the patient during the quiescent phase of the rheumatological disease and, if possible, to plan the vaccination before starting immunosuppression, in particular B cell depleting therapy. Non-live vaccines can also be administered to patients during treatment with systemic glucocorticoids and disease-modifying antirheumatic drugs (DMARDs), while administration of live attenuated vaccines should be considered with caution. In clinical practice, influenza and pneumococcal vaccines are strongly recommended for these patients, while live vaccines, such as yellow fever, should be avoided.

A major concern is that vaccinations may cause an exacerbation or progression of pre-existing autoimmune diseases. The risk/benefit evaluation of recommended vaccines in patients with autoimmune diseases is in favor of vaccination in most cases. Limited data have been published regarding the possibility of vaccine-associated disease exacerbations. For example, several case reports and series have been published on the onset and exacerbation of SLE after HPV vaccination [138,139,140,141], but larger studies have shown neither increased incidence nor exacerbation rate of SLE among vaccinated and unvaccinated patients [142]. The possibility that vaccines could cause or exacerbate multiple sclerosis has been evaluated, but no association was found. In particular, hepatitis B, tetanus, or influenza vaccines did not exacerbate multiple sclerosis [143,144].

## 10. Conclusions

Autoimmune diseases have a complex multifactorial etiology and many factors can contribute to their onset. For this reason, vaccines have also been studied and monitored over time in order to evaluate a possible link between vaccination and the onset of autoimmune diseases or immune-mediated phenomena. However, a causal link between vaccination and AEFI has been ascertained only for a few cases; moreover, AEFI are significantly lower after vaccination than those produced by infection with the wild microorganism, thus confirming the high safety profile of vaccines. Doubts and prejudices about vaccinations can be an obstacle to the achievement of adequate vaccination coverage. In fact, the vaccines have been so successful that many people have never directly experienced the diseases that vaccines prevent. It has been proven that infections contribute to the pathogenesis of autoimmune diseases, while this is very rare for vaccines. For this reason, vaccines have not only the potential to protect the patient from infectious diseases, but also from its complications, including autoimmune manifestations. Nevertheless, it is important to continue AEFI monitoring, especially for new vaccines, such as those developed for SARS-CoV2, in order to evaluate the safety of vaccination, identify any potential signal and maintain confidence in immunization procedures [10,145].

## Data Availability

Not applicable.
